# Spatiotemporal Patterns of Ground Monitored PM_2.5_ Concentrations in China in Recent Years

**DOI:** 10.3390/ijerph15010114

**Published:** 2018-01-11

**Authors:** Junming Li, Xiulan Han, Xiao Li, Jianping Yang, Xuejiao Li

**Affiliations:** 1 School of Statistics, Shanxi University of Finance & Economics, 696 Wucheng Road, Taiyuan 030006, China; Lijunming_dr@126.com (J.L.); inspire_lx@126.com (X.L.); whywhyyjp@163.com (J.Y.); Lixuejiao_ty@126.com (X.L.); 2 LREIS, Institute of Geographic Sciences and Natural Resources Research, Chinese Academy of Sciences, Datun Road 11A, Beijing 10010, China

**Keywords:** PM_2.5_ pollution, Bayeasian statistical model, spatiotemporal patterns

## Abstract

This paper firstly explores the space-time evolution of city-level ${\mathrm{PM}}_{2.5}$ concentrations showed a very significant seasonal cycle type fluctuation during the period between 13 May 2014 and 30 May 2017. The period from October to April following each year was a heavy pollution period, whereas the phase from April to October of the current year was part of a light pollution period. The average monthly ${\mathrm{PM}}_{2.5}$ concentrations in mainland China based on ground monitoring, employing a descriptive statistics method and a Bayesian spatiotemporal hierarchy model. Daily and weekly average ${\mathrm{PM}}_{2.5}$ concentrations in 338 cities in mainland China presented no significant spatial difference during the severe pollution period but a large spatial difference during light pollution periods. The severe ${\mathrm{PM}}_{2.5}$ pollution areas were mainly distributed in the Beijing-Tianjin-Hebei urban agglomeration in the North China Plain during the beginning of each autumn-winter season (September), spreading to the Northeast Plains after October, then later continuing to spread to other cities in mainland China, eventually covering most cities. ${\mathrm{PM}}_{2.5}$ pollution in China appeared to be a cyclic characteristic of first spreading and then centralizing in the space in two spring-summer seasons, and showed an obvious process of first diffusing then transferring to shrinkage alternation during the spring-summer season of 2015, but showed no obvious diffusion during the spring-summer season of 2016, maintaining a stable spatial structure after the shrinkage in June, as well as being more concentrated. The heavily polluted areas are continuously and steadily concentrated in East China, Central China and Xinjiang Province.

## 1. Introduction

Since the beginning of the 21st century, air pollution, especially ${\mathrm{PM}}_{2.5}$ pollution, has caused more than 2 million deaths each year worldwide [1], leading to an increase in hospitalization and mortality rates for asthma and chronic obstructive pulmonary disease (COPD) [2,3]. The research of Pope et al. [4] showed that ${\mathrm{PM}}_{2.5}$ can lead to the deposition of arterial plaque, cause atherosclerosis and vascular inflammation and enhance the risk of cardiovascular disease. When the ${\mathrm{PM}}_{2.5}$ concentration in the air is higher than $10\;\mu g/m^{3}$ for a significant period of time, the risk of death increases significantly, on the basis of $10\;\mu g/m^{3};$ with every additional $10\;\mu g/m^{3}$ increase, the total risk of death increases by 4%, with the risk of death from lung cancer and heart disease increasing by 8% and 6%, respectively [4]. Chen et al. [5] found that prolonged exposure to an environment with an additional $100\;\mu g/m^{3}$ increase of total suspended particulate matter would result in an average reduction of about 3 years in life expectancy [5]. The Global Burden of Disease Study 2010 demonstrated that ${\mathrm{PM}}_{2.5}$ was the ninth most lethal risk factor in the world that year [6]. The death toll caused by ${\mathrm{PM}}_{2.5}$ pollution worldwide increased from 2.91 million in 1990 to 3.22 million in 2010.

Over the past 30 years, China’s economy, society, cities and industries have been undergoing rapid development, which has resulted in increasingly greater energy consumption, particularly coal burning [7]. Simultaneously, rapid urbanization and industrial expansion is proceeding, leading to serious urban ${\mathrm{PM}}_{2.5}$ pollution, probably believed to be a result of traffic-related emissions, industrial emissions, soil dust, biomass burning and regional transported aerosols; however, the mechanism of ${\mathrm{PM}}_{2.5}$ pollution is still not completely understood [8,9]. In mainland China, the annual number of extra deaths caused by air pollution-related diseases is approximately 0.35–0.5 million [10]. The increasing concentration of particulate matter causes the frequency of foggy weather in China to increase continuously and present a clear upward trend [11,12,13,14]; correspondingly, visibility has dropped significantly in most areas since 1990 [11,15,16,17]. The air pollution problem in mainland China has drawn great attention from the government. Since 2013, air quality monitoring has been carried out in 113 key environmental protection cities and environmental protection model cities. In 2015, China established an air quality ground monitoring network covering 338 prefecture-level cities, i.e., sub-provincial administrative divisions, and began releasing reports of average hourly concentrations of air pollutants such as sulfur dioxide (${\mathrm{SO}}_{2}$), nitrogen dioxide (${\mathrm{NO}}_{2}$), carbon monoxide (CO), ozone ($O_{3}$), inhalable particulate matter ${\mathrm{PM}}_{10}$, fine particulate ${\mathrm{PM}}_{2.5}$ and air quality index (AQI) numbers to the public through the China National Urban Air Quality Real-time Publishing Platform [18]. According to recent public reports, to constrain the level of air pollution, the Chinese government has been pursuing various strategies, such as permanently closing the most polluting industrial plants, converting industrial plants from coal to natural gas, and requiring steel, aluminium, plate glass and cement plants to reduce production by up to 50% over the winter period.

Several researchers have studied the problems related to Chinese ${\mathrm{PM}}_{2.5}$ pollution in China. Lin et al. [19] investigated the space-time patterns of ${\mathrm{PM}}_{2.5}$ annual average concentrations in China from 2001 to 2010 based on global annual average ${\mathrm{PM}}_{2.5}$ grids data, and found that the spatial pattern of ${\mathrm{PM}}_{2.5}$ annual concentrations remained stable between 2001 and 2010. Peng et al. [20] systematically explored the spatiotemporal variations of ${\mathrm{PM}}_{2.5}$ annual average concentrations in China from 1999 to 2011 using remotely sensed ${\mathrm{PM}}_{2.5}$ data produced by van Donkelaar et al. [21] in 2015. Li et al. [22] and Lu et al. [23] analyzed the spatiotemporal variations of ${\mathrm{PM}}_{2.5}$ pollution in China from 1998 to 2014 using the latest version of remotely sensed ${\mathrm{PM}}_{2.5}$ annual concentrations data provided by van Donkelaar et al. [24]. Ma et al. [25] estimated the ${\mathrm{PM}}_{2.5}$ annual concentrations from 2004 to 2013 in China using MODIS remote sensing data and described the spatiotemporal trends. This paper presents the first study of the space-time patterns over China in recent years based on in-situ monitoring ${\mathrm{PM}}_{2.5}$ hourly concentration data.

On a countrywide scale in China, previous studies focused on the long-term evolution of ${\mathrm{PM}}_{2.5}$ annual average concentrations based on remotely sensed annual average ${\mathrm{PM}}_{2.5}$ concentrations. Although Zhang & Cao [9] explored Chinese ${\mathrm{PM}}_{2.5}$ pollution based on ground readings, the study period was only one year and not all 338 cities were covered. To our knowledge, this paper is the first to investigate the spatiotemporal variations of ${\mathrm{PM}}_{2.5}$ pollution over China including all 338 cities based on daily average concentrations from May 2014 to May 2017. A Bayesian spatiotemporal hierarchy model (BSTHM) is first employed to estimate the stable spatial patterns of ${\mathrm{PM}}_{2.5}$ concentrations for each month.

## 2. Data and Methodology

### 2.1. Data and Study Area

**Figure 1:**
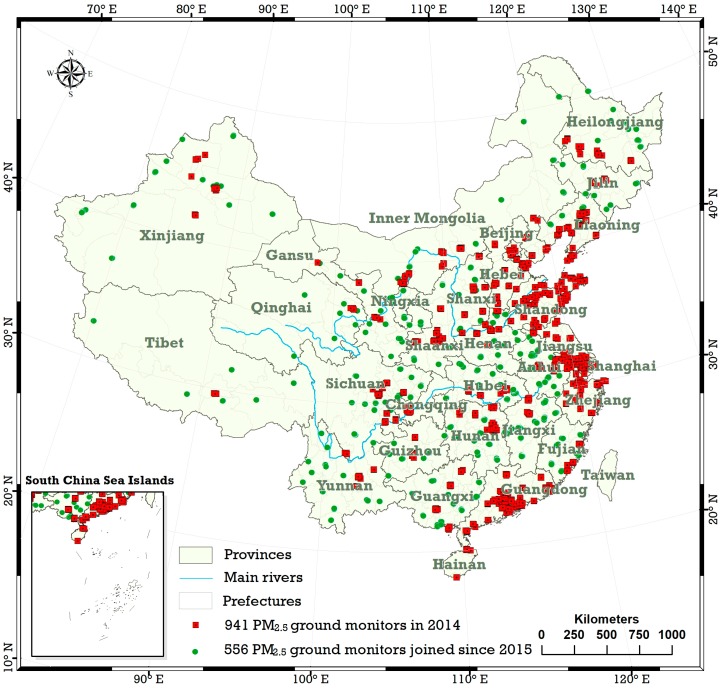
The distribution of air quality monitoring stations in mainland China, with red dots representing the 941 monitoring sites in 2014, and green dots representing the 556 additional sites added in 2015.

The raw data used in this paper is from ground station monitoring ${\mathrm{PM}}_{2.5}$ hourly concentrations from 13 May 2014 to 30 May 2017, as released by the China National Urban Air Quality Real-time Publishing Platform (http://106.37.208.233:20035/). There were 941 monitoring sites from 13 May 2014 to 31 December 2014, with the number increasing to 1497 after 31 December 2014. The site distribution is shown in Figure 1. Table 1 lists the number of ${\mathrm{PM}}_{2.5}$ ground monitoring sites in 31 provinces of mainland China in 2014, 2015, 2016 and 2017. The study area in this paper is mainland China, not including Taiwan. The four municipalities, Beijing, Shanghai, Tianjin and Chongqing, and the other 334 sub-provincial cities, totalling 338 cities, serve as the statistical units. Since 2015, all 338 cities have established ground monitoring stations, with a maximum and minimum number of monitoring stations at 1 and 20, respectively.

The on-site readings of ${\mathrm{PM}}_{2.5}$ concentrations are the most authoritative data and are made public in real time. Random errors can also be eliminated to some extent by average statistical calculation. Moreover, Bayesian statistical estimates themselves can consider more uncertainties by regarding parameters as random variables.

Before conducting statistical analysis, the raw data need to be pre-processed. Because the ${\mathrm{PM}}_{2.5}$ readings from the ground monitoring stations are hourly concentrations, first, the average daily monitoring data, which are the basic inputs for classic and Bayesian statistics, needed to be produced. Each site is assigned a serial number denoting the city where the site distributes. Then the daily, weekly and monthly average ${\mathrm{PM}}_{2.5}$ concentrations of the 338 cities can be obtained. The missing monitoring site data values are filled in using the spatial adjacent-averaging calculation with a 3×3 pixel scope when the classic statistical analysis is conducted; however, they remain missing as null input in the Bayesian statistical estimating process.

### 2.2. Methodology

#### 2.2.1. Mathematical Model Form

This paper’s empirical analysis is based on a BSTHM [26], which is a combination of the Bayesian hierarchy model and a spatiotemporal interaction model. This model offers a comprehensive consideration of the overall spatiotemporal evolution process, and decomposes the coupled spatio-temporal process into three sub-processes of overall spatial effect, overall time effect and temporal-spatial interaction effect (local variation trend). Spatiotemporal observational phenomena usually do not match the two preconditions required in classic statistics—a large sample and independent identical distribution (i.i.d.). First, it is not possible to repeatedly sample in one spatial location at one time point during a space-time process. Second, ‘Tobler’s First Law of Geography’ [27] indicates that spatiotemporal correlation is neither independent nor identically distributed. Therefore, in theory, the inference results for spatiotemporal data based on classic statistics are not reliable. Fortunately, Bayesian statistics does not require a large sample and i.i.d. data as preconditions. The BSTHM can effectively solve the problem of a small sample in a spatiotemporal phenomenon and can make full use of spatiotemporal correlation by utilising prior information. Theoretically speaking, the BSTHM has not only better reliability but is superior to classic statistical models, considering a greater number of uncertainties and being able to generate abundant results. The BSTHM framework generally includes three parts:

(1) $$y_{i}\left|\theta_{j},\Theta \sim P(y_{i}\right|\theta_{j},\Theta )$$

(2) $$\theta_{j},\Theta \sim P(\theta_{j},\Theta |y_{i})$$

(3) Θ~P(Θ)

where $\theta_{j}$ denotes parameters, Θ is hyperparameters, and P(Θ) is hyperprior. According to the Bayesian theorem, $P\left(\theta_{j},{\Theta |y}_{i}\right)$ is the posterior distribution of parameters and hyperparameters, and the formula can be expressed as:

(4) $$P\left(\theta_{j},{\Theta |y}_{i}\right)\propto P(y_{i}|\theta_{j},\Theta )P\left(\theta_{j},\Theta \right)$$

For the research described in this paper, the data from the monitoring station is the observation value, the 338 cities are the spatial statistical units and the corresponding mathematical expression is as follows:

(5) $$y_{it}\sim N\left(\mu_{c\left[i\right],t},\sigma_{y}^{2}\right)$$

(6) $$\ln\left(\mu_{c\left[i\right],t}\right)=\alpha +S_{c\left[i\right]}+\left(b_{0}t^{*}+v_{t}\right)+b_{c\left[i\right],t}t^{*}+\varepsilon_{c\left[i\right],t}$$

where $y_{it}$ is the ${\mathrm{PM}}_{2.5}$ monitoring concentration of i(i=1,2,…,1497) station at time t, c[i] is the serial number of the city where *i* monitoring station is located, $\mu_{c\left[i\right],t}$ is the ${\mathrm{PM}}_{2.5}$ concentration of c[i] city at time *t*, $\sigma_{y}^{2}$ is the variance, α represents the common level of ${\mathrm{PM}}_{2.5}$ pollution over the Chinese mainland in the study period, $S_{c\left[i\right]}$ is the overall spatial relativity parameter of c[i] city, $b_{o}t^{*}+v_{t}$ describes the overall trend, which consists of a linear trend $b_{o}t^{*}$ (allow nonlinear trend) and a random effect $v_{t}$, $b_{c\left[i\right]}$ is the local trend of c[i] city. $\varepsilon_{c\left[i\right],t}$ is a Gaussian noise random variable capturing additional variability in the data not explained by other variables in the model.

More specifically, each month will be a study sub-period, then the common spatial component will be coagulated from the coupled space-time evolution process considering the overall time trend $b_{o}t^{*}+v_{t}$ and local trend $b_{c\left[i\right],t}t^{*}$. Furthermore, the estimated posterior median of $\exp\left(S_{c\left[i\right]}\right)$ measures a relative magnitude of the ${\mathrm{PM}}_{2.5}$ pollution level, a ratio of the level of c[i] city to the common level over the Chinese mainland, exp(α).

#### 2.2.2. Determination of Prior Distribution

This paper employs the Besag York Mollie (BYM) model [28], which is a convolution of a spatially structured and unstructured random effect to determine the prior distribution of the process model parameters $S_{c\left[i\right]}$ and $b_{c\left[i\right]}.$ Spatial structure is imposed by the conditional auto-regression (CAR) prior with a first-order spatial adjacency matrix W, where its diagonal entries are $w_{ij}=0$ and the off-diagonal entries are $w_{ij}=1$ if the spatial statistical units i and j share a common boundary, and $w_{\mathrm{ij}}=0$ otherwise. In other words, the spatial correlation is established according to the topological relation of the multiscale homogeneous subdivided grids. The mathematical expression is:

(7) $$l\left(y|\theta ,\Theta \right)=\underset{it\in S_{it}}{\prod }f(y_{it}|\theta_{it},\Theta )$$

where l(y|θ,Θ) is sample likelihood function, $S_{it}$ is space-time domain, $y_{it}$ are observed sample values, $|\theta_{it}$ are the spatiotemporal process variables, and Θ is the hyperparameter set. Here, the conditional auto regression (CAR) prior distribution is used to represent the random effects of spatial structure. The spatial adjacency matrix W adopts the first-order “queen” adjoining form. Gaussian noise is $\varepsilon_{\mathrm{it}}\sim N\left(\sigma_{\varepsilon }^{2}\right)$. According to the conclusion of Gelman [29], the prior distribution of mean square error (such as $\sigma_{v}$, $\sigma_{\varepsilon }$) for all random variables in the model is determined as a strictly positive half-Gaussian distribution $N_{+\infty }\;\left(0,\;10\right)$.

In this paper, Bayesian estimation is achieved using WinBUGS [30], which is specifically for Bayesian statistics based on the Markov Chain Monte Carlo (MCMC) method [31,32]. Two MCMC chains are used to ensure the convergence and reliability of the estimation results. The number of iterations for each chain is set to 250,000, of which, 200,000 are for the burn-in period and 50,000 are for the number of iterations of posterior distribution of parameters. The reliability of Bayesian statistical inference is assessed by convergence. The convergence of the Bayesian statistical results in this paper is evaluated with the Gelman-Rubin statistical parameter estimation, in which the closer the value is to 1, the better the convergence is [33]. The Gelman-Rubin parameters of all parameters in this study range from 0.99 to 1.01, indicating that the convergence of these statistical results is steady, or the Bayesian statistical estimated results are reliable.

## 3. Results

### 3.1. Descriptive Statistics

**Figure 2:**
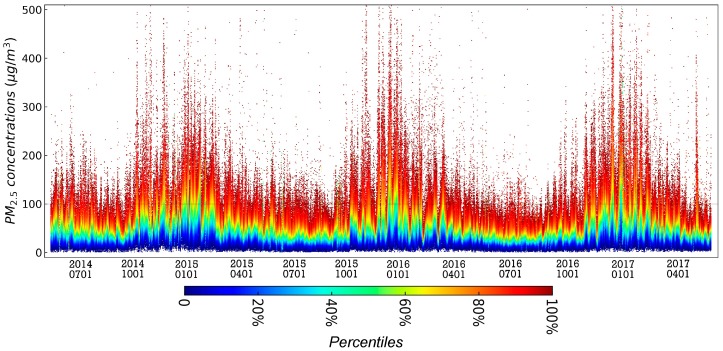
The time series chart of the ground monitor station date of daily average ${\mathrm{PM}}_{2.5}$ concentration from 13 May 2014 to 30 May 2017 in mainland China. The color represents the percentile.

Figure 2 is a time series chart of the percentiles of ${\mathrm{PM}}_{2.5}$ daily average concentrations monitored by the ground monitor stations in mainland China from 13 May 2014 to 30 May 2017. The daily ${\mathrm{PM}}_{2.5}$ average concentration is calculated based on the hourly average concentration. As can be seen from Figure 2, ${\mathrm{PM}}_{2.5}$ daily average concentration showed a clear seasonal fluctuation in general, with the period from October to April of the next year belonging to the heavy pollution period, while the period from April to October of the same year belonged to the light pollution period. During the entire study period, some monitoring stations had ${\mathrm{PM}}_{2.5}$ concentrations higher than $100\;\mu g/m^{3}$ each day, especially during the heavy pollution period. More than 50% of the ground monitoring stations’ ${\mathrm{PM}}_{2.5}$ concentrations were higher than $100\;\mu g/m^{3}$, and some even exceeded 300 $\mu g/m^{3}$. In addition, throughout the study period, the quantile below 20% maintained a relatively stable state, while the quantile above 50% showed a distinctive seasonal jump characteristic.

**Figure 3:**
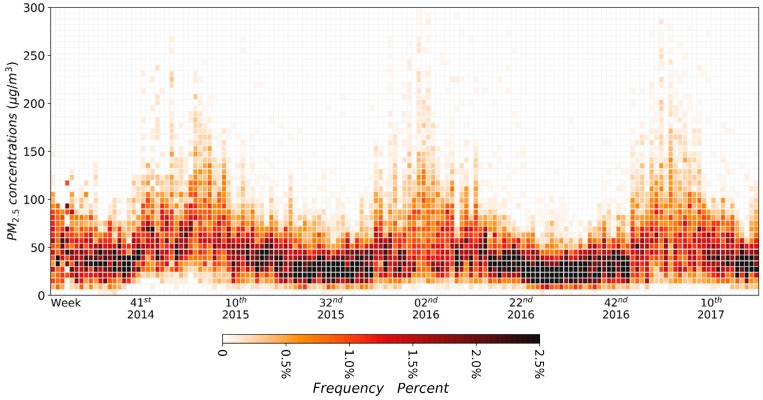
The time series thermodynamic histogram of ${\mathrm{PM}}_{2.5}$ weekly average concentration distribution in 338 cities in mainland China.

In order to study the variation characteristics of ${\mathrm{PM}}_{2.5}$ pollution in 338 cities in mainland China during the study period, this paper draws a time series thermodynamic histogram of ${\mathrm{PM}}_{2.5}$ weekly average concentration in Chinese mainland cities, as shown in Figure 3. The distribution of ${\mathrm{PM}}_{2.5}$ weekly average concentration in Chinese mainland cities also showed obvious seasonal fluctuation in the time dimension. The period from the 41st week of the year to the 10th week of the following year was the heavy pollution period, while the period from the 10th to the 41st week of the current year was the light pollution period. The weekly average concentration of ${\mathrm{PM}}_{2.5}$ in Chinese mainland cities was mainly distributed in the range of $0–100\;\mu g/m^{3}$; during the heavy pollution period, the cities with ${\mathrm{PM}}_{2.5}$ weekly average concentration above $100\;\mu g/m^{3}$ appeared. Figure 2 demonstrates that the three severe pollution periods of 2014–2015, 2015–2016 and 2016–2017 showed a tendency of shortening; that is, the number of weeks with ${\mathrm{PM}}_{2.5}$ concentrations higher than $100\;\mu g/m^{3}$ gradually decreased along the three heavy pollution periods, while the ups and downs were becoming “thinner and thinner”.

**Figure 4:**
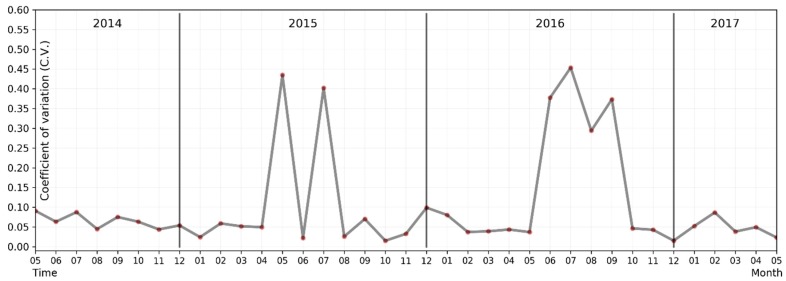
Distribution variation coefficient of ${\mathrm{PM}}_{2.5}$ in Chinese mainland cities from May 2014 to May 2017.

This paper also analysed the spatial heterogeneity of ${\mathrm{PM}}_{2.5}$ monthly average concentration in Chinese mainland cities and its variation based on the spatial variation coefficient (as shown in Figure 4), which is a ratio of the standard variation and mean value of the monthly average ${\mathrm{PM}}_{2.5}$ concentrations for all 338 cities at a cross-section. The higher the variation coefficient, the greater the spatial heterogeneity is. As can be seen in Figure 4, during the period of heavy pollution (from October to January of the following year), there was no significant spatial difference among ${\mathrm{PM}}_{2.5}$ monthly average concentrations in cities of mainland China. While the period of light pollution (from January to October of the same year) involved a large spatial difference, especially in the period from June–September 2016, the spatial variation coefficient continued to rise, with a minimum of 0.29 and a maximum of 0.45. There were also similarly high spatial differences in May and July 2015, with corresponding variation coefficients of 0.44 and 0.43, respectively. The variation coefficient of other months remained below 0.10.

### 3.2. Bayesian Statistics Results

According to the results of the above descriptive statistical analysis, ${\mathrm{PM}}_{2.5}$ pollution in mainland China showed a strong seasonal fluctuation throughout the overall study period. Therefore, when estimates using the Bayesian spatiotemporal model are made, a natural year is divided into two seasons: an autumn-winter season (from September to February of the next year) and a spring-summer season (from March to August of the current year). Then, ${\mathrm{PM}}_{2.5}$ daily average concentration data is used as observational sample data. The steady-state spatial pattern of ${\mathrm{PM}}_{2.5}$ pollution in each of the two seasons is measured using the posterior median of $\exp\left(S_{c\left[i\right]}\right)$ estimated from its posterior probability density. $\exp\left(S_{c\left[i\right]}\right)>1.0$ (<1.0) indicates that ${\mathrm{PM}}_{2.5}$ pollution in c[i] city is $\exp\left(S_{c\left[i\right]}\right)$ times the overall level in mainland China in this month, measured by the parameter exp(α). Since the parameter $\exp\left(S_{c\left[i\right]}\right)$ is derived after decomposing the global and the local trend, it has a steady state characteristic.

#### 3.2.1. The Spatial and Temporal Evolution of the Autumn-Winter Season

**Figure 5:**
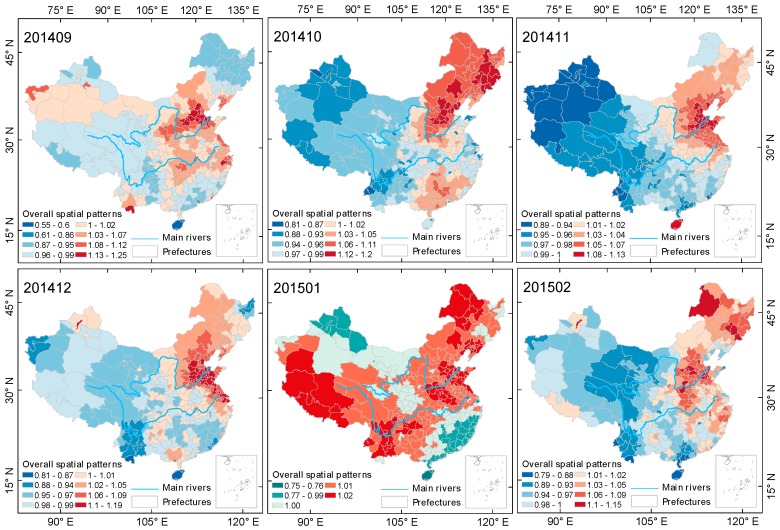
Evolution of spatial relativity of ${\mathrm{PM}}_{2.5}$ pollution over Chinese mainland (not including Taiwan) during the autumn-winter season of 2014–2015.

**Figure 6:**
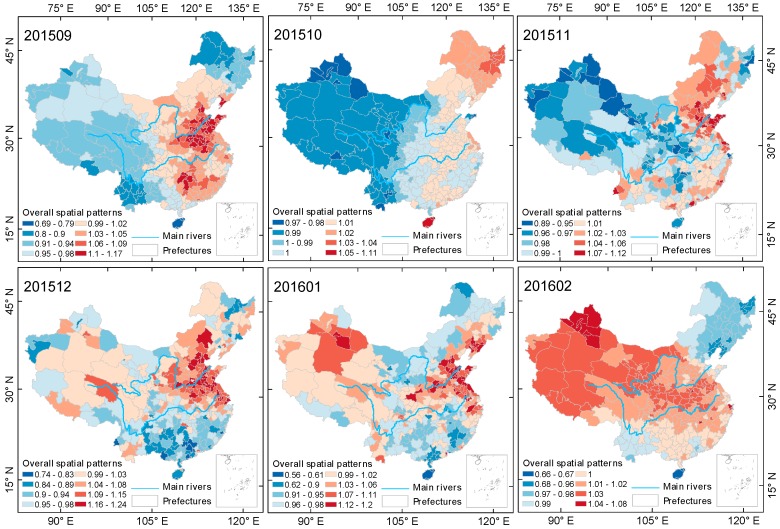
Evolution of spatial relativity of ${\mathrm{PM}}_{2.5}$ pollution over Chinese mainland (not including Taiwan) during the autumn-winter season of 2015–2016.

**Figure 7:**
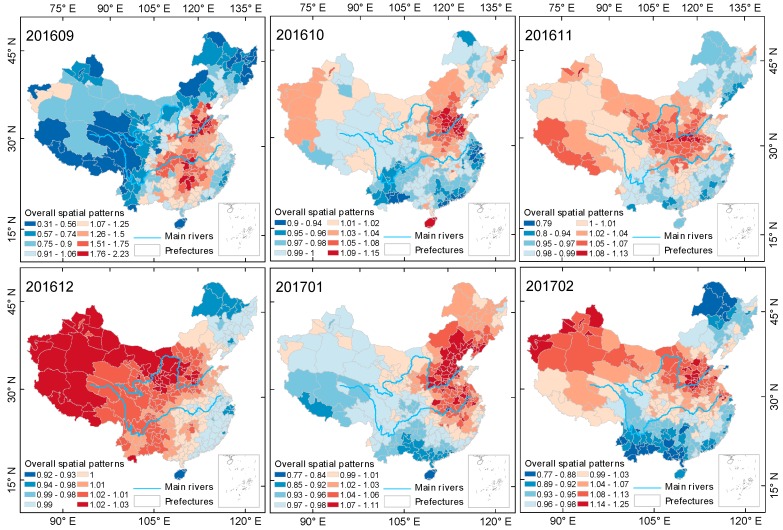
Evolution of spatial relativity of ${\mathrm{PM}}_{2.5}$ pollution over the Chinese mainland (not including Taiwan) during the autumn-winter season of 2016–2017.

Figure 5, Figure 6 and Figure 7 shows the evolution of the steady-state spatial pattern of cities’ ${\mathrm{PM}}_{2.5}$ pollution in mainland China during the autumn-winter seasons from 2014–2017. The results show that in each autumn-winter cycle, the spatial pattern of ${\mathrm{PM}}_{2.5}$ pollution shows different distribution features every month.

In general, the spatial pattern evolution processes of the three autumn-winter cycles have some similarities. In the early part of each autumn-winter season (September), the areas with serious ${\mathrm{PM}}_{2.5}$ pollution are mainly distributed in the Beijing-Tianjin-Hebei urban agglomeration area of the North China Plain. After September, the heavy pollution areas spread to the northeast plains and then to other cities in mainland China, with ${\mathrm{PM}}_{2.5}$ pollution in most of the cities in China reaching more serious levels. However, regardless of month, ${\mathrm{PM}}_{2.5}$ pollution levels in the North China Plain were the highest.

Specific to each autumn-winter cycle, the spatial pattern of ${\mathrm{PM}}_{2.5}$ pollution in mainland China exhibits its own characteristics. In January 2015, February 2016 and December 2016, the spatial differences in ${\mathrm{PM}}_{2.5}$ pollution were smallest, and the corresponding maximum values of steady state spatial pattern coefficients $\exp\left(S_{c\left[i\right]}\right)\;$ were 1.02, 1.08 and 1.03, respectively, which is consistent with the conclusion of the aforementioned variation coefficient and can be specifically justified by the Bayesian spatiotemporal model from a spatial perspective. Figure 2 shows that the above-mentioned months are the periods of most severe pollution, that is, most of the cities in mainland China during those three months were experiencing severe ${\mathrm{PM}}_{2.5}$ pollution. In addition, during the autumn-winter season of 2014–2015, with the exception of January 2015, the areas with heavy ${\mathrm{PM}}_{2.5}$ pollution were mostly concentrated in the eastern and northeastern areas north of Shanghai and Zhejiang. During the last two autumn-winter cycles, ${\mathrm{PM}}_{2.5}$ pollution in western cities began to increase. Over three months (December 2015, January 2016 and February 2016) of the 2015–2016 autumn-winter season and over four months (October 2016, November 2016, December 2016, and February 2017) of the 2016–2017 autumn-winter season, a situation emerged in which ${\mathrm{PM}}_{2.5}$ pollution levels in the western cities were higher than the overall levels were. In the three autumn-winter cycles, ${\mathrm{PM}}_{2.5}$ pollution in the northeastern region had a tendency to decrease gradually. In the first, second and third autumn-winter seasons, the months in which ${\mathrm{PM}}_{2.5}$ pollution in the northeastern region was lower than the overall level were 1, 3 and 4, respectively.

#### 3.2.2. The Spatial and Temporal Evolution of the Spring-Summer Season

**Figure 8:**
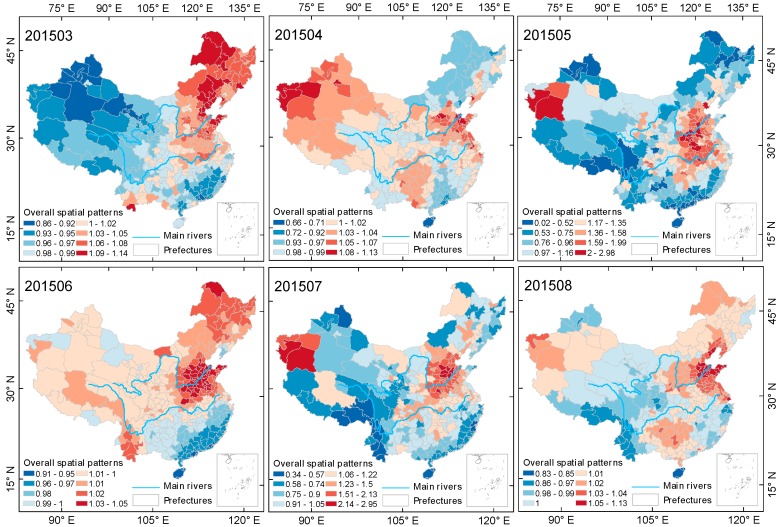
Evolution of spatial relativity of ${\mathrm{PM}}_{2.5}$ pollution over the Chinese mainland (not including Taiwan) during the spring-summer season of 2015.

**Figure 9:**
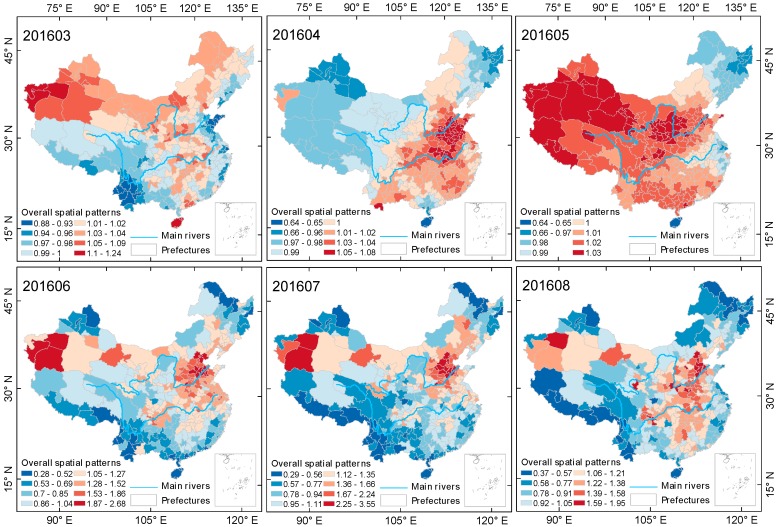
Evolution of spatial relativity of ${\mathrm{PM}}_{2.5}$ pollution over the Chinese mainland (not including Taiwan) during the spring-summer season of 2016.

As the ground monitor site data used in this paper is from 13 May 2014 to 30 May 2017, the spring-summer seasons of the years 2014 and 2017 are incomplete. This section selects two complete spring-summer seasons, that is, the data from 2015 and 2016, for research. Figure 8 and Figure 9 show the evolution process of spatial pattern of ${\mathrm{PM}}_{2.5}$ pollution in Chinese mainland cities during two spring-summer seasons, estimated by the Bayesian time-space model. ${\mathrm{PM}}_{2.5}$ pollution in China showed a cyclic characteristic of first spreading and then centralizing in space during both seasons, and showed an obvious process of first diffusing then transferring to shrinkage alternation during the spring-summer of 2015, but no obvious diffusion during the spring-summer season of 2016, maintaining a stable spatial structure after the shrinkage in June.

From the results based on the Bayesian spatiotemporal model estimation, we can see that the spatial difference of ${\mathrm{PM}}_{2.5}$ pollution in mainland China was more significant during the spring-summer season than the autumn-winter season. In May and July 2016 and June and July 2016, the spatial relativity coefficient $\mathrm{EXP}\left(S_{c\left[i\right]}\right)$ reached 2.98 and 2.95, and 2.68 and 3.55, respectively. Most of the heavy pollution areas in the above months were located in the Beijing-Tianjin-Hebei urban agglomeration in the North China Plain, with some heavy pollution areas also located in Xinjiang. During the spring-summer cycle of 2016, heavily polluted areas expanded. However, the degree of pollution in the northeastern region showed a decreasing trend in both annual spring-summer seasons, with the number of months with ${\mathrm{PM}}_{2.5}$ pollution lower than the overall level being 2 and 5, respectively. The spatial distribution of ${\mathrm{PM}}_{2.5}$ pollution in mainland China was more concentrated during the spring-summer season in 2016, with the heavily polluted areas continuously and steadily concentrated in East China, Central China and Xinjiang.

#### 3.2.3. Trend Analysis

**Figure 10:**
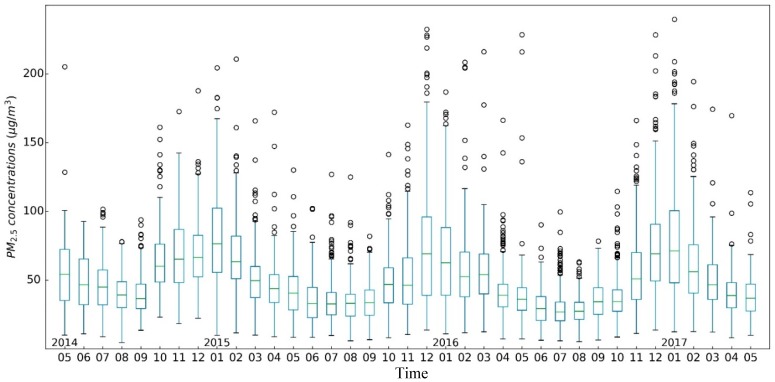
Box-plot chart of ${\mathrm{PM}}_{2.5}$ monthly average concentration in Chinese mainland cities from May 2014 to May 2017.

${\mathrm{PM}}_{2.5}$ pollution in Chinese cities exhibited different trend characteristics during different seasons. During the spring-summer seasons of 2015 and 2016, ${\mathrm{PM}}_{2.5}$ monthly average concentrations in Chinese mainland cities showed a month to month downward trend (Figure 10), and in most cities, ${\mathrm{PM}}_{2.5}$ monthly average concentration was maintained at a level of less than $100\;\mu g/m^{3}$. During the autumn-winter seasons of 2015, 2016 and 2017, ${\mathrm{PM}}_{2.5}$ monthly average concentrations showed a trend of first increasing and then decreasing. In August of three autumn-winter seasons, ${\mathrm{PM}}_{2.5}$ monthly average concentrations in the Chinese mainland were the lowest, and then continued to increase. In January 2015, December 2015 and January 2017, ${\mathrm{PM}}_{2.5}$ pollution in the cities of mainland China reached the highest levels for each respective year. ${\mathrm{PM}}_{2.5}$ monthly average concentrations in 25% of cities (85 cities) exceeded $100\;\mu g/m^{3}$, and the median of each autumn-winter season was $76.46\;\mu g/m^{3}$, $69.08\;\mu g/m^{3}$ and $71.18\;\mu g/m^{3}$, respectively. According to Figure 5, Figure 6 and Figure 7, the spatial distribution of heavily polluted cities in the highly polluted months showed a gradually concentrating trend from 2015 to 2017. The number of cities with ${\mathrm{PM}}_{2.5}$ pollution exceeding the national average was 231 in January 2015 (only the southeastern Guangdong and Fujian and a small part of northern Xinjiang were excluded), which was reduced to 163 in December 2015 (mainly including cities in East China, North Central China and the western regions (Xinjiang, Qinghai and Tibet)) and then increased to 174 in January 2017 (mostly concentrated in east China and northeast China).

## 4. Discussion

This paper researches the space-time evolution process of ${\mathrm{PM}}_{2.5}$ pollution in mainland China in recent years, employing the descriptive statistics method and the BSTHM based on ${\mathrm{PM}}_{2.5}$ hourly concentration data from ground monitoring stations over the past three years. Most other research on the temporal and spatial patterns of ${\mathrm{PM}}_{2.5}$ pollution is based on the large scale nation-level, and on the ${\mathrm{PM}}_{2.5}$ annual average concentration data, which is an inversion in remote sensing. However, the reliability of remote sensing inversion data of ${\mathrm{PM}}_{2.5}$ concentration is apparently weaker than the ground monitoring data, and its time span is usually in annual units, rendering the granularity too large to explore many fine temporal and spatial evolution characteristics and laws. Moreover, the ${\mathrm{PM}}_{2.5}$ daily, weekly and monthly average concentrations are more closely related to the public environment and public health, and corresponding research is therefore more practical and instructive.

The results of a series of studies based on ${\mathrm{PM}}_{2.5}$ annual average concentration of remote sensing inversion data [19,20,34] indicate that the spatial pattern of ${\mathrm{PM}}_{2.5}$ pollution at the annual scale will generally maintain a certain steady state structure. However, this study shows that the ${\mathrm{PM}}_{2.5}$ pollution spatial pattern at the fine-grained time scale has very unsteady characteristics, as the spatial distribution of ${\mathrm{PM}}_{2.5}$ pollution in mainland China presents significant differences during different months of different seasons. Through this fine-grained study in time and space, it is possible to develop a deeper understanding of the evolution of regional ${\mathrm{PM}}_{2.5}$ pollution and make policy formulation more pertinent to relevant environmental governance.

${\mathrm{PM}}_{2.5}$ pollution is an environmental threat to China as well as to other developing countries, such as India and Saudi Arabia. As outlined above, the Chinese government has been taking active measures to combat ${\mathrm{PM}}_{2.5}$ pollution. The statistical results in this paper show that some success has been realised, however, there is still a great deal of room for air quality improvements in China. ${\mathrm{PM}}_{2.5}$ pollution is not just a local issue—it can be considered a countrywide or even world-wide phenomenon. To control ${\mathrm{PM}}_{2.5}$ pollution, the Chinese government needs to consider spatial and temporal heterogeneity simultaneously. Resource management and policy formulation should target ${\mathrm{PM}}_{2.5}$ pollution treatment in specific regions and months. This paper provides detailed spatial city-level patterns for each month. Meanwhile, macroscopic national and urban regional policy implications can be provided. Precise measures not only result in fine effects and save limited resource, but they also minimally influence public lifestyle, for example, not having to impose regular traffic restrictions. In addition, at present, the ${\mathrm{PM}}_{2.5}$ pollution over China in the winter heating season is being given greater attention. Nevertheless, ${\mathrm{PM}}_{2.5}$ pollution in the other seasons—autumn, spring and summer—cannot be overlooked. The phenomenon of ${\mathrm{PM}}_{2.5}$ pollution is also dependent upon a time axis. Therefore, policies for preventing and remedying ${\mathrm{PM}}_{2.5}$ pollution should be formulated according to its monthly evolution over China. In so doing, the results achieved through conformance to policies would be ultimately more effective.

## 5. Conclusions

This paper presents the first systematic study of the space-time pattern evolution process of ${\mathrm{PM}}_{2.5}$ pollution in mainland China over the most recent three years based on available ground monitoring station data. The study findings are: (i) ${\mathrm{PM}}_{2.5}$ pollution in mainland China showed a very significant seasonal cycle type fluctuation, with the period from October to April following a previous year being a heavy pollution period, while the phase from April to October of the current year belonged to the light pollution period. Throughout the entire study period, there was a situation of ${\mathrm{PM}}_{2.5}$ concentration higher than $100\;\mu g/m^{3}$ every day, especially during the heavy pollution period. It was noted that over 50% of the monitoring stations’ ${\mathrm{PM}}_{2.5}$ daily average concentration levels were higher than $100\;\mu g/m^{3}$; (ii) The three severe pollution periods of 2014–2015, 2015–2016 and 2016–2017 demonstrated a shortening tendency, meaning that the number of weeks with ${\mathrm{PM}}_{2.5}$ concentration levels higher than $100\;\mu g/m^{3}$ gradually decreased over the three heavy pollution periods. The average monthly concentration of ${\mathrm{PM}}_{2.5}$ in the cities of mainland China presented no significant spatial differences during the severe pollution period (from October to January of the next year) and but a large spatial difference during the light pollution period (from January to October of the same year); (iii) The severe ${\mathrm{PM}}_{2.5}$ pollution areas were mainly distributed in the Beijing-Tianjin-Hebei urban agglomeration in the North China Plain at the beginning of each autumn-winter season (September), and spread to the Northeast Plains after October, then continued to spread to other cities in mainland China later, eventually covering most of the cities in China. Regardless of month, however, ${\mathrm{PM}}_{2.5}$ pollution in the North China Plain was the highest; (iv) ${\mathrm{PM}}_{2.5}$ pollution in China showed a cyclic characteristic of proliferation-contraction in space during two spring-summer seasons, showing an obvious process of diffusion (transfer)-shrinkage alternation in the spring-summer season of 2015 but no obvious diffusion in the spring-summer season of 2016, maintaining a stable spatial structure after the shrinkage in June and being more concentrated. The heavily polluted areas are continuously and steadily concentrated in east China, central China and Xinjiang province; (v) the spatial distribution of heavily polluted cities in the highly polluted months showed a gradually concentrated trend from 2015 to 2017. The number of cities with ${\mathrm{PM}}_{2.5}$ pollution exceeding the national average was 231 in January 2015 (only southeastern Guangdong, Fujian and a small part of northern Xinjiang were excluded), a number reduced to 163 in December 2015 (mainly including east China, north central China and the western regions (Xinjiang, Qinghai and Tibet)) before increasing to 174 in January 2017 (mostly concentrated in east China and northeast China).

However, there are some specific shortcomings of this paper. First, only ${\mathrm{PM}}_{2.5}$ was selected from the ground station monitoring data pertaining to pollutants, meaning other air pollutants such as ${\mathrm{SO}}_{2}$ and ${\mathrm{NO}}_{2}$ were not included in the study. If all of the pollutants monitored by the ground stations were to be included in the research, more comprehensive results would be obtained. Second, this paper uses the urban administrative divisions as the spatial units and uses ${\mathrm{PM}}_{2.5}$ concentration data monitored by the sites within the city areas to describe the ${\mathrm{PM}}_{2.5}$ pollution levels of each city. Therefore, the estimations based on considering city areas as the relevant spatial units may be biased due to the unbalanced layout of the ground monitoring stations (most of them are located in urban areas, with only a small number of control sites being located in the outskirts). In the next phase, we will break down the urban administrative division and use other space division units for further study.

## Figures and Tables

**Table 1:** The number of ${\mathrm{PM}}_{2.5}$ ground monitoring sites in 31 provinces in mainland China in 2014, 2015, 2016 and 2017.

Province Name	Number of Sites in 2014	Number of Sites in 2015, 2016, and 2017	Added Number of Sites
Beijing	12	12	0
Tianjin	14	14	0
Hebei	53	53	0
Shanxi	32	59	27
Inner Mongolia	23	44	21
Liaoning	62	79	17
Jilin	17	33	16
Heilongjiang	27	57	30
Shanghai	9	9	0
Jiangsu	97	97	0
Zhejiang	55	55	0
Anhui	19	68	49
Fujian	13	37	24
Jiangxi	17	60	43
Shandong	100	100	0
Henan	37	75	38
Hubei	18	51	33
Hunan	39	78	39
Guangdong	102	103	1
Guangxi	22	50	28
Hainan	7	7	0
Chongqing	17	17	0
Sichuan	40	94	54
Guizhou	15	33	18
Yunnan	12	40	28
Tibet	6	18	12
Shaanxi	37	50	13
Gansu	10	33	23
Qinghai	4	11	7
Ningxia	10	19	9
Xinjiang	15	41	26
Total	941	1497	556
